# A Comprehensive Analysis of the Dynamic Biological Networks in HCV Induced Hepatocarcinogenesis

**DOI:** 10.1371/journal.pone.0018516

**Published:** 2011-04-19

**Authors:** Bing He, Hao Zhang, Tieliu Shi

**Affiliations:** 1 Center for Bioinformatics and Computational Biology, The Institute of Biomedical Sciences, School of Life Science, East China Normal University, Shanghai, China; 2 Department of General Surgery, Huashan Hospital, Fudan University, Shanghai, China; 3 Shanghai Information Center for Life Sciences, Chinese Academy of Science, Shanghai, China; Health Canada, Canada

## Abstract

Hepatocellular carcinoma (HCC) is a primary malignancy of the liver, which is closely related to hepatitis C and cirrhosis. The molecular mechanisms underlying the hepatocarcinogenesis induced by HCV infection remain clarified from a standpoint of systems biology. By integrating data from protein-protein interactions, transcriptional regulation, and disease related microarray analysis, we carried out a dynamic biological network analysis on the progression of HCV induced hepatocarcinogenesis, and systematically explored the potentially disease-related mechanisms through a network view. The dysfunctional interactions among proteins and deregulatory relationships between transcription factors and their target genes could be causes for the occurrence and progression of this disease. The six pathologically defined disease stages in the development and progression of HCC after HCV infection were included in this study. We constructed disease-related biological networks for each disease stage, and identified progression-related sub-networks that potentially play roles in the developmental stage of the corresponding disease and participate in the later stage of cancer progression. In addition, we identified novel risk factors related to HCC based on the analysis of the progression-related sub-networks. The dynamic characteristics of the network reflect important features of the disease development and progression, which provide important information for us to further explore underlying mechanisms of the disease.

## Introduction

Hepatocellular carcinoma (HCC) is a major health problem worldwide. It is the sixth most common neoplasm in the world with more than half a million new cases annually [1], and the main cause of death among cirrhotic patients [2]. Hepatocarcinogenesis is a complex and multistep process, which is associated with many risk factors [3]. Hepatitis B virus (HBV) and hepatitis C virus (HCV) infections, excessive alcohol consumption and aflatoxin are widely recognized as the four major etiological factors of HCC [4]. Chronic infection with hepatitis B virus is the predominant risk factor for HCC in Southeast Asia and Africa, while chronic infection with hepatitis C virus is the predominant risk factor for HCC in Western countries and Japan.

The risk of HCC in patients with chronic hepatitis C is the highest and has been well studied in patients who have established cirrhosis [5], [6], [7], [8]. The incidence of HCC in patients with cirrhosis is between 2%–8% per year as reported based on clinical studies. A previous study showed that [9] 12,008 HCV-positive men conferred a 20-fold increased risk of HCC compared to those HCV-negative subjects. However, the presence or absence of cirrhosis was not evaluated. It was reported that HCV-infected individuals without cirrhosis had a much lower risk of developing HCC [10]. Although previous clinically-based studies revealed close relationships among hepatitis C, cirrhosis and HCC, the underlying molecular mechanism of these phenomena remained unclear.

As the center for the diversified metabolisms and detoxification, liver plays various functions through different parenchymal cell types. Each type of cell is composed of thousands of different types of molecules. A discrete biological function can only rarely be attributed to an individual molecule. In contrast, most biological functions arise from interactions among many components. Usually, ‘modules’ are used to describe this kind of biological organization. Modules are composed of different types of molecules. They have discrete functions that arise from interactions among their components (proteins, DNA, RNA and small molecules), but these functions cannot easily be predicted by studying the properties of the isolated components [11]. Transcriptional regulatory interaction and protein-protein interaction are two of the most important interactions among these components. Transcriptional regulation is the process in which gene-encoded transcription factors regulate the transcription of other genes. The assembly of regulatory interactions linking transcription factors to their target genes in an organism can be viewed as a directed graph, in which the nodes represent the regulators and their targets, and the regulatory interactions are the edges. Protein-protein interactions (PPIs) are crucial for all biological processes [12]. Previous study on protein–protein interaction networks in liver cancer detected many interactions among proliferation, apoptosis-related proteins and differential glycoproteins, suggesting that a “molecule groups” concept should be introduced in the diagnosis and metastasis prediction of HCC instead of analyzing a single, or a few proteins [13]. In all, because the majority of gene products function together with other gene products, biological processes should be considered as complex networks of interconnected components. In other words, for any biological process, one might consider a ‘modular approach’ in which the behavior and function of the corresponding network are studied as a whole, in addition to studying some of its components individually [11].

Recently, the innovation of high throughput experimental strategies makes it possible for biology to become holistic [14]. With the tremendous increase in human protein interaction data, network approaches have already been employed to understand molecular mechanisms of diseases [15], particularly to analyze carcinogenesis and the genotype related to the cancer phenotype. However, most efforts only focused on protein-protein interaction network. Moreover, few of these efforts studied dynamic changes of the network during the development and progression from pre-HCC disease to HCC, which will facilitate us to understand the molecular mechanisms of hepatocarcinogenesis.

Our hypothesis is that the dysfunctional genes/proteins and interactions among them at each progression-related sub-network are strongly related to the special disease pathogenic state, and play a role in the disease progression. Networks related to each stage of the disease can capture the pathogenic characteristics of corresponding disease stages and reflect the role of those genes/proteins in the development and progression of hepatocarcinogenesis at the disease specific stage. Based on this hypothesis, we have integrated multiple data at different levels, especially with regard to biological pathways and interaction networks, and built a hepatocellular carcinoma biological network database previously [16]. The integrated network lays the foundation for our current work. With the integration of protein-protein interactions data, transcriptional regulatory interactions (TRIs) data and microarray data covering hepatitis C, cirrhosis and Barcelona Clinic Liver Cancer (BCLC) staging HCC, we carried out a dynamic biological network analysis for the progression of HCV induced hepatocarcinogenesis.

## Results

### Biological network and interaction annotation

In our previous work, we have integrated a set of human protein-protein interaction data and transcriptional regulatory interaction data, which contains 37811 experimentally confirmed protein-protein interactions and 9148 experimentally confirmed transcriptional regulatory interactions [16]. These interactions form a molecular biological network in humans. Gene Ontology is commonly used for annotation of genes and proteins [17]. However, the function of a module in a network is not only determined by a group of individual molecules, but also the functional association between them. Therefore, annotating the function of these interactions in a biological network will facilitate us to understand systemic functions in the biological network. A transcription factor may achieve its function by regulating the expression of target genes that implement the same function. In this way, if a transcription factor and its target gene could be mapped to a same function annotation, the TRI between them may also participate in this function. Based on Gene Ontology annotation data, which is supported by experimental evidence, we annotated interactions of the human biological network. Finally, 21365 PPIs and 3587 TRIs were annotated by this method.

### Disease-related biological network

Biological networks provide valuable information for understanding cellular function and biological processes. The dysfunction of some interactions causes many diseases, including cancers. Therefore, studying the deregulated biological networks will help us to understand molecular mechanisms of these diseases. First, we identified DEGs (Differentially Expressed Genes) by bioinformatics analysis of the microarray data that cover Hepatitis C, Cirrhosis, and BCLC staging HCC. Then we mapped those DEGs to protein-protein interactions data and transcriptional regulatory interactions data integrated in HCCNet database [16], which was a HCC network database previously established by our team. In each disease stage, the PPI between DEGs-encoded proteins, and the TRI between the DEG-encoded transcription factor and its target gene in DEGs were regarded as deregulated. By integrating these interactions, we constructed disease-related biological networks that were deregulated in each disease stage (see details in material and method).

**Figure 1:**
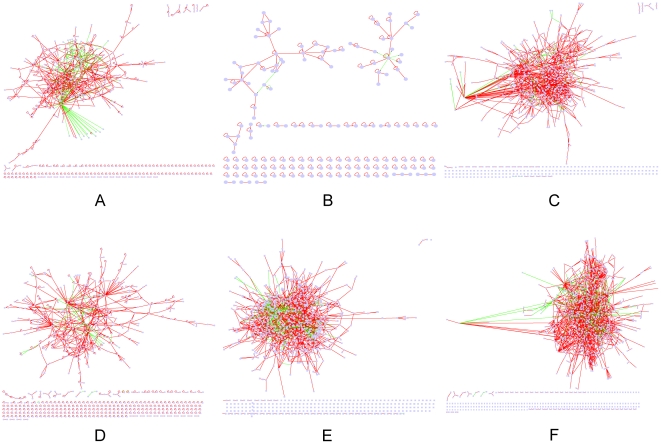
Disease related biological networks. Edge color: red, PPI; green, TRI. Edge direction: undirected, PPI; directed, TRI, from transcriptional factor to target gene. (A) Hepatitis related biological network; (B) cirrhosis related biological network; (C) BCLC stage 0 HCC related biological network; (D) BCLC stage A HCC related biological network; (E) BCLC stage B HCC related biological network; (F) BCLC stage C HCC related biological network.

Several classification systems are available for HCC. The Barcelona Clinic Liver Cancer (BCLC) classification has emerged during recent years as the standard classification that is used for trial design and clinical management of patients with HCC [18], [19]. It divides HCC into five stages. Patients with end-stage disease (stage D) have a reported median survival of only 3 months [18]. Therefore, this stage is excluded in the following study. Six disease stages in the development and progression of HCV induced hepatocarcinogenesis are included in our study including Hepatitis C, Cirrhosis, BCLC stage 0 HCC, BCLC stage A HCC, BCLC stage B HCC, BCLC stage C HCC. We identified disease-related biological networks for each disease stage with the process described above (Figure 1). The deregulated biological network of hepatitis C was constructed, bearing 605 nodes and 999 edges, while the deregulated biological network of cirrhosis included 166 nodes and 197 edges. The deregulated biological network of BCLC stage 0 HCC was composed of 883 nodes and 1545 edges. Meanwhile, the deregulated biological network of BCLC stage A HCC contained 734 nodes and 1057 edges. The deregulated biological network of BCLC stage B HCC was generated with 1053 nodes and 1783 edges. The deregulated biological network of BCLC stage C HCC was constructed by 1277 nodes and 2378 edges (Supplementary Data S1). In these networks, edges represent PPIs or TRIs. Line in PPI is undirected when line in TRI is directed. Nodes linked by the edge in PPI are proteins. The edge in TRI goes from transcription factor to its target gene.

### Dynamic deregulated biological network

Comparison among deregulated biological networks shows that when the disease progression switches sequentially from hepatitis C to BCLC stage C HCC, the deregulated biological network changes correspondingly. The dynamic network implies the potential molecular mechanism that underlies the development and progression of the carcinogenesis. Some interactions were deregulated consistently in different stages during the disease development and progression. We hypothesize that these shared interactions participate in the development and progression of the carcinogenesis. For example, an interaction that was deregulated in hepatitis C initially and remained dysfunctional in the following cirrhosis and at all the stages of HCC may participate in the hepatitis C infection, and the development and progression from hepatitis C to BCLC stage C HCC.

**Figure 2:**
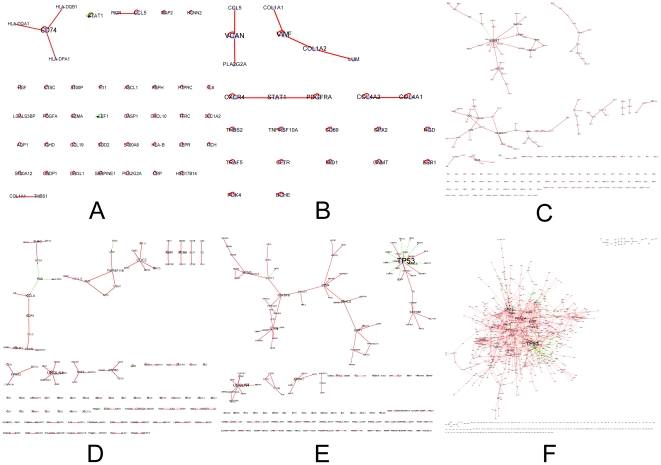
Sub networks identified in developing progression of HCV induced hepatocarcinogenesis. Edge color: red, PPI; green, TRI. Edge direction: undirected, PPI; directed, TRI, from transcriptional factor to target gene. (A) sub networks that were deregulated initially from hepatitis; (B) the ones that were deregulated initially from cirrhosis; (C) the ones that were deregulated initially form BCLC stage 0 HCC; (D) the ones that were deregulated initially form BCLC stage A HCC; (E) the ones that were deregulated initially form BCLC stage B HCC; (F)the ones that were deregulated initially form BCLC stage C HCC.

Based on the above hypothesis, we made a comparison among the six disease-related biological networks in the development and progression. 42 interactions were deregulated initially from hepatitis C to BCLC stage C HCC. 26 interactions were deregulated initially from cirrhosis, which means that they were not deregulated in hepatitis C but in cirrhosis, BCLC stage 0 HCC, BCLC stage A HCC, BCLC stage B HCC and BCLC stage C HCC. Accordingly, 224 interactions showed deregulated only from BCLC stage 0 HCC when 114 interactions were deregulated initially from BCLC A HCC. Furthermore, 156 interactions were indentified deregulated initially from BCLC B HCC and 1186 interactions were recognized as deregulated initially from BCLC C HCC (Supplementary Data S2). By integrating interactions that were deregulated initially from the same stage, progression-related sub-networks, which contains fifty six sub-networks (node> = 3), were constructed (Figure 2).

### Functional annotation of progression-related sub-networks

In our hypothesis, genes and proteins in a progression-related sub-network may function together in the progression of HCV induced hepatocarcinogenesis. To better understand their functions in the progression, especially the role of the sub-network in the progression, we need to find out which function is significant comparing to the whole human biological network. Gene Ontology enrichment analysis is usually used to identify statistically significant functions of a group of genes. However, this method treats those genes as individual components and ignores their association, which is the base of a module. We have annotated integrated human biological networks with Gene Ontology data, which is supported by experimental evidence. Based on hypergeometric distribution, we carried out a function enrichment analysis between the progression-related sub-network and the whole human biological network. Statistically significant functions (p value<0.01) of the progression-related sub-networks, which indicate the functional difference comparing to the whole biological network, were identified through this analysis. In the total number of fifty six sub-networks, twenty seven of them were identified with statistically significant functions (Supplementary Data S3). The genes and proteins in the progression-related sub-network identified with statistically significant functions may work together in the progression of HCV induced hepatocarcinogenesis. Furthermore, the statistically significant functions of the progression-related sub-network indicate the functions these genes and proteins perform by synergistic action.

We offered these progression-related sub-networks and their statistically significant functions in the supplementary materials. In our hypothesis, the sub-network that is deregulated from hepatitis C is considered a cirrhosis-HCC-risk sub-network. The dysfunction of it could increase the risk of cirrhosis and following HCC incidence of hepatitis C patients. Many functions that related to cirrhosis and cancer are significantly enriched in this sub-network, such as cell proliferation, positive regulation of fibroblast proliferation, negative regulation of apoptosis, etc. (Table 1). The sub-networks which are deregulated from cirrhosis can be regarded as HCC-risk sub-networks. The dysfunction of them could increase HCC incidence for hepatitis C patients with established cirrhosis. Some functions related to cancer are overrepresented in these sub-networks, like cell adhesion, positive regulation of DNA replication, positive regulation of cell proliferation, etc (Supplementary Data S3).

**Table 1:** Significant functions (P value<0.01) of the progression-related sub network deregulated initially from hepatitis C.

GO ID	P value	Name
**GO:0050731**	1.28E-03	positive regulation of peptidyl-tyrosine phosphorylation
**GO:0004896**	1.70E-04	cytokine receptor activity
**GO:0030890**	5.96E-04	positive regulation of B cell proliferation
**GO:0001961**	1.70E-04	positive regulation of cytokine-mediated signaling pathway
**GO:0043518**	5.11E-04	negative regulation of DNA damage response, signal transduction by p53 class mediator
**GO:0008283**	1.11E-03	cell proliferation
**GO:0016021**	1.96E-03	integral to membrane
**GO:0001516**	3.41E-04	prostaglandin biosynthetic process
**GO:0002792**	1.70E-04	negative regulation of peptide secretion
**GO:0019955**	3.41E-04	cytokine binding
**GO:0001540**	5.96E-04	beta-amyloid binding
**GO:0007165**	3.66E-03	signal transduction
**GO:0048146**	0.00127703	positive regulation of fibroblast proliferation
**GO:0043066**	0.004082191	negative regulation of apoptosis
**GO:0005773**	0.000170341	Vacuole
**GO:0009986**	0.005609786	cell surface
**GO:0070374**	0.000766365	positive regulation of ERK1 and ERK2 cascade

**Figure 3:**
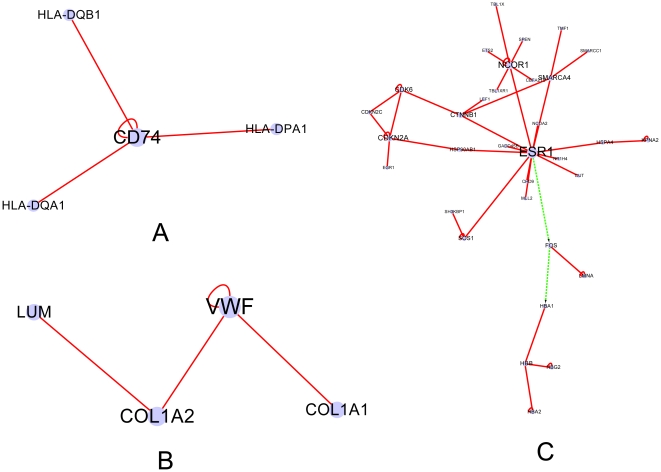
Sub networks that mentioned in discussion. (A) Sub network that deregulated initially from hepatitis C. (B) One of the sub networks that deregulated initially from cirrhosis; (C) One of the sub networks that deregulated initially from BCLC stage 0 HCC.

To verify our hypothesis and those identified disease relate sub-networks. We randomly selected three sub-networks from the twenty seven progression-related sub-networks annotated as statistically significant functions and carried out the text mining from published literature. One sub-network is deregulated from hepatitis C, another sub-network is generated from cirrhosis data and the last one signifies the deregulation from BCLC stage 0 HCC. In the sub-network that is deregulated from hepatitis C (Figure 3A), HLA-DQA1 and DQB1 are associated with development of cirrhosis, and DQB1 could be risk factors for the occurrence of HCC [20], [21]. COL1A2, a protein in the sub-network which is deregulated from cirrhosis, is involved in the development or progression of hepatoma [22]. Moreover, ESR1, the core protein, which interacts with most nodes in this sub-network, of the network that is deregulated from BCLC stage 0 HCC, have been shown to be associated with an increased hepatocellular carcinoma risk [23].

## Discussion

The progression-related sub-network deregulated in hepatitis C infection stage was constructed with four nodes (HLA-DQA1, HLA-DQB1, HLA-DPA1 and CD74) and interactions among them (Figure 3A). We proposed that this sub-network is potentially related to cirrhosis-HCC-risk, considering that those genes are differentially expressed in hepatitis C stage. It could be seen that many important functions, including positive regulation of B cell proliferation, negative regulation of apoptosis, etc., are enriched significantly (P value<0.01) in this sub-network (Table 1). In this sub-network, CD74 is the major histocompatibility complex, class II invariant chain. HLA-DQA1 and HLA-DPA1 belong to the HLA class II alpha chain paralogues while HLA-DQB1 belongs to the HLA class II beta chain paralogues.

The class II molecule is a heterodimer consisting of an alpha (DPA, DQA) and a beta (DPB, DQB) chain, both anchored in the membrane. It plays a central role in the immune system by presenting peptides derived from extracellular proteins. Within the DP and DQ molecules both the alpha chain and the beta chain contain the polymorphisms specifying the peptide binding specificities, resulting in up to 4 different molecules.

DQB1*0201 allele has been confirmed to be closely correlated with the progression of liver injury in chronic HCV infection [24]. Meanwhile, DQA1*0103 appears to provide protection against chronic active infection with hepatitis C virus [25]. Moreover, HLA-DQA1 and DQB1 are associated with development of cirrhosis, and DQB1 might be a risk factor for the occurrence of HCC [20], [21]. Since full function of major histocompatibility complex needs both the invariant chain and variant chain, the PPIs among DQA1, DQB1 and CD74 may participate in the induction of hepatitis C and the development and progression from hepatitis C to HCC.

Our network-based view shows that three progression-related sub-networks appear deregulated initially in cirrhosis stage. One of the sub-networks is composed of four nodes (VWF, LUM, COL1A1 and COL1A2) with four interactions among them (Figure 3B), and it can be considered an HCC-risk related sub-network. Certain functions, like, cell adhesion, are significantly enhanced (P value<0.01) in this sub-network (Table 2). Within the same sub-network, VWF functions as an antihemophilic factor carrier and a platelet-vessel wall mediator in the blood coagulation system. LUM is a member of the small leucine-rich proteoglycan (SLRP) family that includes decorin, biglycan, fibromodulin, keratocan, epiphycan, and osteoglycin. In these bifunctional molecules, the protein moiety binds collagen fibrils and the highly charged hydrophilic glycosaminoglycans regulate interfibrillar spacings. Lumican is the major keratan sulfate proteoglycan of the cornea but is also distributed in interstitial collagenous matrices throughout the body. Lumican may regulate collagen fibril organization and circumferential growth, corneal transparency, and epithelial cell migration and tissue repair. COL1A1 is the pro-alpha1 chains of type I collagen whose triple helix comprises two alpha1 chains and one alpha2 chain. COL1A2 is the pro-alpha2 chain of type I collagen whose triple helix comprises two alpha1 chains and one alpha2 chain. Type I is a fibril-forming collagen found in most connective tissues and is abundant in bone, cornea, dermis and tendon.

**Table 2:** Significant functions (P value<0.01) of the progression-related sub network deregulated initially from cirrhosis.

GO ID	P value	Name
**GO:0005783**	5.86E-03	endoplasmic reticulum
**GO:0007596**	4.26E-04	blood coagulation
**GO:0047485**	5.02E-03	protein N-terminus binding
**GO:0033093**	1.70E-04	Weibel-Palade body
**GO:0019865**	1.70E-04	immunoglobulin binding
**GO:0030168**	6.81E-04	platelet activation
**GO:0051087**	8.51E-04	chaperone binding
**GO:0051260**	1.70E-03	protein homooligomerization
**GO:0005178**	1.11E-03	integrin binding
**GO:0005576**	7.57E-07	extracellular region
**GO:0007599**	1.70E-04	Hemostasis
**GO:0031012**	6.81E-04	extracellular matrix
**GO:0043231**	0.003232767	intracellular membrane-bounded organelle
**GO:0002020**	0.001362122	protease binding
**GO:0031589**	0.000170341	cell-substrate adhesion
**GO:0007155**	0.000766365	cell adhesion
**GO:0005518**	0.000510959	collagen binding
**GO:0001948**	0.000936609	glycoprotein binding

COL1A1 and COL1A2 are associated with liver fibrogenesis [26], [27]. Moreover, COL1A2 is involved in the development or progression of hepatoma [22]. VWF mRNA has been shown to be significantly upregulated in both fibrosis and HCC [28]. In patients with fulminant hepatic failure and liver cirrhosis, circulating plasma VWF antigen levels are extremely high [29], [30], [31]. Many fibrin thrombi have been found in the hepatic sinusoids in acute liver failure, suggesting a role for intravascular coagulation in the pathogenesis of hepatic necrosis [32]. In cirrhotic liver tissue [33] and even tissue from patients in early stages of alcoholic liver diseases [34], VWF immunostaining shows positive cells predominantly at the scar–parenchyma interface, within the septum, and in the sinusoidal lining. Portal or hepatic vein thrombosis is often observed in advanced cirrhosis [35], [36] and microthrombi formation has been found in one or multiple organs in half of autopsied cirrhotics [37]. This hypercoagulable state in liver diseases may be involved in hepatic parenchymal extinction, the acceleration of liver fibrosis, and disease progression. Lumican has diverse biologic roles but has been thought to be primarily involved in fibrosis of the extracellular matrix through the binding of collagen fibrils and regulation of their lateral growth [38], [39]. Lumican expression is also increased with progression of hepatic fibrosis in rats [40]. Decreased sulforylation of lumican side chains stimulates macrophage adhesion and the cellular inflammatory response [41], [42], suggesting that changes in the structure of lumican may promote the inflammatory process that precedes and enhances collagen deposition during the process of hepatic fibrosis. Taken together, it implies that PPIs among COL1A1, COL1A2, VWF and LUM may participate in the induction of cirrhosis and play roles in the progression from cirrhosis to HCC.

The progression-related sub-networks deregulated from BCLC stage 0 HCC included 11 sub-networks. The biggest sub-network in this category was comprised of 34 nodes with 42 PPIs and 2 TRIs (Figure 3C). Many functions are significantly enriched in this sub-network (Table 3), including induction of apoptosis, cell cycle arrest, etc. A core protein of this sub-network is ESR1, which interacts with most nodes in this sub-network. This protein regulates the expression of FOS, and subsequently FOS regulates HBA1. HBA1 participate in a smaller sub-network that constructed by four nodes (HBA1, HBB, HBA2, HBG2) with interactions among them. ESR1 is an estrogen receptor, a ligand-activated transcription factor composed of several domains important for hormone binding, DNA binding, and activation of transcription. FOS can dimerize with proteins of the JUN family, thereby forming the transcription factor complex AP-1. As such, the FOS proteins have been implicated as regulators of cell proliferation, differentiation, and transformation. In some cases, expression of the FOS gene has also been associated with apoptotic cell death. The alpha (HBA) and beta (HBB) loci determine the structure of the 2 types of polypeptide chains in adult hemoglobin, Hb A. The normal adult hemoglobin tetramer consists of two alpha chains and two beta chains. Mutation in beta globin is the cause of sickle cell anemia. Absence of beta chain causes beta-zero-thalassemia. Reduced amounts of detectable beta globin causes beta-plus-thalassemia.

**Table 3:** Significant functions (P value<0.01) of the progression-related sub network deregulated initially from BCLC stage 0 HCC.

GO ID	P value	Name
**GO:0000118**	9.14E-08	histone deacetylase complex
**GO:0005634**	3.22E-07	Nucleus
**GO:0005876**	7.34E-07	spindle microtubule
**GO:0005515**	2.34E-06	protein binding
**GO:0017053**	2.75E-06	transcriptional repressor complex
**GO:0003714**	5.82E-06	transcription corepressor activity
**GO:0004861**	8.55E-06	cyclin-dependent protein kinase inhibitor activity
**GO:0042326**	1.79E-05	negative regulation of phosphorylation
**GO:0010553**	2.92E-05	negative regulation of gene-specific transcription from RNA polymerase II promoter
**GO:0000082**	0.000129787	G1/S transition of mitotic cell cycle
**GO:0005737**	4.66E-04	Cytoplasm
**GO:0042826**	7.90E-04	histone deacetylase binding
**GO:0007050**	0.00090131	cell cycle arrest
**GO:0006917**	9.79E-04	induction of apoptosis
**GO:0030308**	0.001019554	negative regulation of cell growth
**GO:0008285**	1.23E-03	negative regulation of cell proliferation
**GO:0019901**	0.001821703	protein kinase binding
**GO:0030332**	1.87E-03	cyclin binding
**GO:0043697**	0.001871365	cell dedifferentiation
**GO:0045646**	0.001871365	regulation of erythrocyte differentiation
**GO:0042063**	0.001871365	Gliogenesis
**GO:0007517**	0.001871365	muscle organ development
**GO:0046825**	0.001871365	regulation of protein export from nucleus
**GO:0005088**	0.001871365	Ras guanyl-nucleotide exchange factor activity
**GO:0008637**	0.001871365	apoptotic mitochondrial changes
**GO:0071158**	0.001871365	positive regulation of cell cycle arrest
**GO:0044428**	1.87E-03	nuclear part
**GO:0045668**	2.81E-03	negative regulation of osteoblast differentiation
**GO:0070215**	2.81E-03	MDM2 binding
**GO:0043517**	0.002805763	positive regulation of DNA damage response, signal transduction by p53 class mediator
**GO:0010389**	0.002805763	regulation of G2/M transition of mitotic cell cycle
**GO:0001953**	0.002805763	negative regulation of cell-matrix adhesion
**GO:0031648**	2.81E-03	protein destabilization
**GO:0007265**	0.003255111	Ras protein signal transduction
**GO:0051225**	3.74E-03	spindle assembly
**GO:0045736**	3.74E-03	negative regulation of cyclin-dependent protein kinase activity
**GO:0001954**	0.003739306	positive regulation of cell-matrix adhesion
**GO:0004693**	4.67E-03	cyclin-dependent protein kinase activity
**GO:0000080**	0.004671996	G1 phase of mitotic cell cycle
**GO:0032993**	0.004671996	protein-DNA complex
**GO:0000075**	5.60E-03	cell cycle checkpoint
**GO:0006338**	0.005603832	chromatin remodeling
**GO:0006309**	0.005603832	DNA fragmentation involved in apoptosis
**GO:0050680**	0.006534815	negative regulation of epithelial cell proliferation
**GO:0046329**	0.007464947	negative regulation of JNK cascade
**GO:0050821**	0.007464947	protein stabilization
**GO:0032088**	8.39E-03	negative regulation of NF-kappaB transcription factor activity
**GO:0016055**	0.008394228	Wnt receptor signaling pathway
**GO:0006469**	0.009322658	negative regulation of protein kinase activity
**GO:0051059**	9.32E-03	NF-kappaB binding

Animal models and human epidemiologic studies have suggested that estrogens act as tumor promoters and might induce hepatocarcinogenesis [43], [44], [45], [46]. The estrogens exert the effects by binding to estrogen receptors (ESR). The genetic polymorphisms within ESRs could influence the effects of estrogens, which in turn results in genotype-dependent differences in risk for hepatocellular carcinoma. Indeed, the polymorphisms in the 5′ end of the ESR a (ESR1) gene have been shown to be associated with an increased hepatocellular carcinoma risk, supporting the involvement for the estrogen-ESR axis in the estrogen-induced hepatocarcinogenesis [23]. The oncogene c-fos (FOS), which is required for quiescent cells to enter the cell cycle [47], also is up-regulated in HCC [48]. HBV X peptide has been shown to activate the c-fos gene, which is postulated to contribute to hepatocarcinogenesis [49]. Our result implies that the association between ESR1 and FOS may participate in the process of HCC initiation and may play roles in the progression of carcinogenesis from early to advanced HCC. Moreover, the hemoglobin (Hb A) level may be deregulated by this association in the progression of carcinogenesis from early HCC.

**Figure 4:**
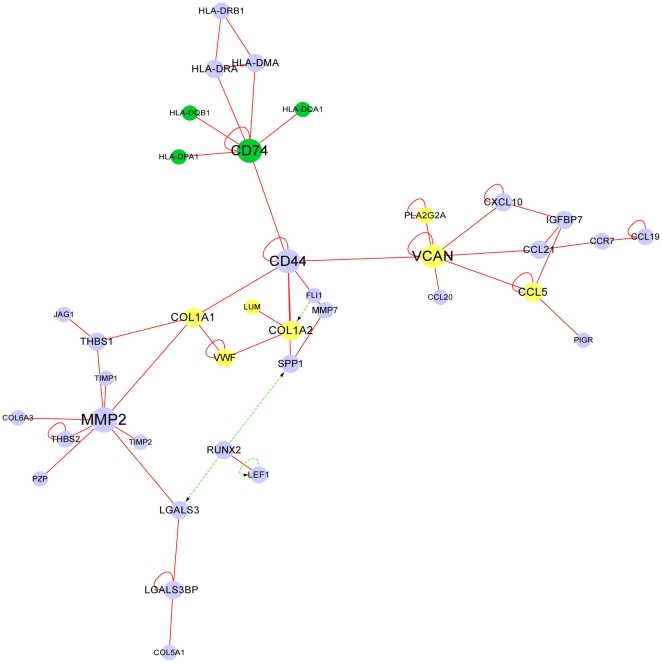
Cirrhosis-related network mentioned in discussion. Node color: green, nodes used to be in cirrhosis-HCC-risky sub-network; yellow, nodes in HCC-risky sub-network; purple, other nodes in cirrhosis-related network. Edge color: red, PPI; green, TRI. Edge direction: undirected, PPI; directed, TRI, from transcriptional factor to target gene.

Previous studies have indicates that when hepatitis C patients establishes cirrhosis, the HCC incidence will increase largely. Cirrhosis is an important stage in the progression from hepatitis C to HCC. In the cirrhosis-related network, we can recognize some interesting components and relationships (Figure 4). The nodes that appeared in cirrhosis-HCC-risk sub-network are located close to the nodes belonging to HCC-risk sub-networks. They are connected by CD44. The CD44 is a cell-surface glycoprotein. The CD74, a protein of cirrhosis-HCC-risky related sub-network, is also membrane protein and the rest of the sub-network proteins are located extracellularly. Similarly, the proteins in HCC-risk related sub-networks are also located in extracellular region. It could be anticipated that the synergistic action between deregulated cirrhosis-HCC-risky related sub-network and HCC-risky related sub-networks could be one of the reasons for the dramatic increase of HCC incidence when hepatitis C patients develop cirrhosis. As we mentioned before, the dysfunction of cirrhosis-HCC-risk related sub-network emerges from hepatitis C, while the dysfunction of HCC-risk related sub-networks gets started from cirrhosis. Those relationships and results supported by the experimental evidences imply that the dysfunction of HCC-risk related sub-networks is under the influence of the dysfunction of the cirrhosis-HCC-risk related sub-network.

Although we have identified progression-related sub-networks for HCV induced hepatocarcinogenesis and recognized some potential synergistic actions of proteins and genes in the sub-network, our approach has certain limitations. First, we only use experimentally confirmed interaction data in our analysis. So some potential associations among DEGs may be lost. Second, our analysis depends not only on biological reality but also on sampling, experimental conditions, and reported results; there could be other genes and proteins that significantly contribute to network functions not yet addressed.

In conclusion, we constructed the disease-related biological networks through the integration of those DEGs, PPIs and TRIs data for different stages of hepatitis C infected disease, including liver cancer. By comparison of the disease-related biological networks between each stage, the dynamic characteristics of the networks show that they mostly reflect the important features of the disease development and progression, which provides important information for us to explore the underlying mechanisms of the diseases. We identified progression-related sub-networks in the development and progression through dynamic biological network analysis and annotated significant functions of these sub-networks. Text mining results from published literature confirmed our hypothesis largely in the examples randomly chosen. It implies that these progression-related sub-networks, especially the ones which are annotated with significant functions, can be helpful in the understanding of molecular mechanism that underlies the progression of HCV induced hepatocarcinogenesis.

## Materials and Methods

### Microarray data and DEG

Microarray data was collected from Gene Expression Omnibus (GEO) database. Two datasets (GSE6764, GSE9843) were used in this analysis. They contain 10 normal samples, 21 hepatitis samples, 13 cirrhosis samples, 9 BCLC stage 0 HCC samples, 56 BCLC stage A HCC samples, 7 BCLC stage B HCC samples, 8 BCLC stage C HCC samples. Normal samples, hepatitis samples and cirrhosis samples were from GSE6764. BCLC staging HCC samples were from GSE9843. All samples except for normal ones were HCV infected. Both GSE6764 and GSE9843 were based on GPL570 platform: [HG-U133_Plus_2] Affymetrix Human Genome U133 Plus 2.0 Array. And there were no technical replicates in both of the two datasets. The differentially expressed genes (DEGs) were collected for further analysis if the expression alteration between the disease sample and normal controls was greater than 2 fold. We identified up-regulated DEGs and down-regulated DEGs for each disease sample.

### Detection of deregulated biological network

Protein-protein interactions data and transcriptional regulatory interactions data came from HCCNet database [16], which was a HCC network database previously established by our team, The database contains 37811 experimentally confirmed protein-protein interactions and 9148 experimentally confirmed transcriptional regulatory interactions. Those data were used to construct deregulated biological networks of each disease stage. Firstly, DEGs indentified in each disease sample were mapped to PPI and TRI data. Then deregulated interactions between DEGs were identified and used to construct the individual deregulated biological network. An individual deregulated biological network is a network constructed by deregulated PPIs and TRIs identified in one disease sample. When all individual deregulated networks of a disease were constructed, occurrence frequency of each deregulated interaction in the disease could be calculated. 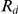 represents the occurrence frequency of a deregulated interaction in a disease. 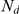 signifies the number of disease samples in which the interaction is deregulated. 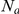 represents the number of all the samples of the disease.
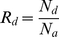
(1)If 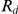 of an interaction is more than 0.5, it means that this interaction is deregulated in more than half of the cases of a disease, the interaction can be regarded as high confidently deregulated one and the dysfunction value X of the interaction is defined as 1, otherwise it is defined as 0. In other words, we believe those interactions whose X value equals to 1 are deregulated in the disease.
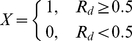
(2)With those indentified deregulated interactions, deregulated biological network of each disease stag was constructed for further analysis. Finally, deregulated biological networks in six stages were established accordingly.

### Detection of progression-related biological sub-network

When the progression of the disease advances from hepatitis C to BCLC stage C HCC, the deregulated biological network changes correspondingly. Six disease stages were considered in this progression: Hepatitis C, Cirrhosis, BCLC stage 0 HCC, BCLC stage A HCC, BCLC stage B HCC, BCLC stage C HCC. Some interactions are deregulated initially in one stage and keep dysfunction in the following stages, they may participate in the development stage of the disease in which they are deregulated initially and may also play a role in the following progression process. As defined above, if an interaction is deregulated in a disease stage, the X value of the interaction is 1, otherwise the X value is 0. So when an interaction is deregulated initially in hepatitis C and keeps dysfunction in the following disease stage, the 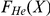 will be 1. In other words, when the value of 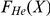 is 1, the interaction is initially in hepatitis C and keeps dysfunction in the following disease stage.
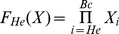
(3)Generally, we defined an indicator function F_S_(X) as:
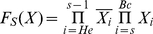
(4)which equals to 1 when an interaction is deregulated initially in stage *S* and keeps dysfunction in the following disease stages, where 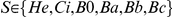. *He* represents hepatitis C, while Ci represents cirrhosis. Similarly, B0, Ba, Bb and Bc represent BCLC stage 0, BCLC stage A, BCLC stage B and BCLC stage C of HCC, respectively. The order is from *He* to *Bc*. So when *S* is Ci, S-1 is He. When S is *He*, we define S-1 is 0 and 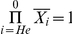. In other words, when *S* is *He*, the formula can be presented as: 
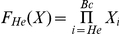
.

### Detection of significant functions of sub-networks

In our previous work, we annotated each interaction of integrated biological network with a new Gene Ontology based annotation method that developed by ourself. Gene Ontology annotation data of *Homo sapiens* was downloaded on April 20, 2010. We filtered annotations which are supported by experiment evidence and used them in the annotation of interactions. With the annotated interaction data and hypergeometric distribution method, we compare each sub-network to the whole biological network and identified statistical significant functions (p value<0.01) of each sub-network. Statistical analysis is performed with R.

## Supporting Information

Data S1 Disease-related biological networks in each stage of HCV induced hepatocarcinogenesis.
(XLS)

Data S2 Progression-related sub networks in the progression of HCV induced hepatocarcinogenesis.
(XLS)

Data S3 Functional annotation of progression-related sub networks in the progression of HCV induced hepatocarcinogenesis.
(TXT)
